# Tobacco smoking confers risk for severe COVID‐19 unexplainable by pulmonary imaging

**DOI:** 10.1111/joim.13190

**Published:** 2020-12-03

**Authors:** J. Li, X. Long, Q. Zhang, X. Fang, N. Li, B. Fedorova, S. Hu, Jh. Li, N. Xiong, Z. Lin

**Affiliations:** ^1^ From the Medical Treatment Expert Group for COVID‐19 Wuhan Red Cross Hospital Wuhan Hubei China; ^2^ Department of Neurology Union Hospital Tongji Medical College Huazhong University of Science and Technology Wuhan Hubei China; ^3^ Department of Radiology Union Hospital Tongji Medical College Huazhong University of Science and Technology Wuhan Hubei China; ^4^ Department of Emergency Medicine Sana‐Klinikum Offenbach Hessen Germany; ^5^ Department of Radiology Wuhan Red Cross Hospital Wuhan Hubei China; ^6^ Department of Medicine University of California San Diego La Jolla CA USA; ^7^ McLean Hospital Harvard Medical School Belmont MA USA

**Keywords:** addiction, epidemiology, lung damage, SARS‐CoV‐2, substance use disorders

## Abstract

**Background:**

COVID‐19 is a new pneumonia. It has been hypothesized that tobacco smoking history may increase severity of this disease in the patients once infected by the underlying coronavirus SARS‐CoV‐2 because smoking and COVID‐19 both cause lung damage. However, this hypothesis has not been tested.

**Objective:**

Current study was designed to focus on smoking history in patients with COVID‐19 and test this hypothesis that tobacco smoking history increases risk for severe COVID‐19 by damaging the lungs.

**Methods and results:**

This was a single‐site, retrospective case series study of clinical associations, between epidemiological findings and clinical manifestations, radiographical or laboratory results. In our well‐characterized cohort of 954 patients including 56 with tobacco smoking history, smoking history increased the risk for severe COVID‐19 with an odds ratio (OR) of 5.5 (95% CI: 3.1–9.9; *P* = 7.3 × 10^−8^). Meta‐analysis of ten cohorts for 2891 patients together obtained an OR of 2.5 (95% CI: 1.9–3.3; *P* < 0.00001). Semi‐quantitative analysis of lung images for each of five lobes revealed a significant difference in neither lung damage at first examination nor dynamics of the lung damage at different time‐points of examinations between the smoking and nonsmoking groups. No significant differences were found either in laboratory results including D‐dimer and C‐reactive protein levels except different covariances for density of the immune cells lymphocyte (*P* = 3.8 × 10^−64^) and neutrophil (*P* = 3.9 × 10^−46^).

**Conclusion:**

Tobacco smoking history increases the risk for great severity of COVID‐19 but this risk is achieved unlikely by affecting the lungs.

## Graphical abstract

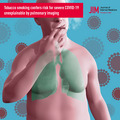

## Introduction

The highly contagious severe acute respiratory syndrome coronavirus 2 (SARS‐CoV‐2) has infected more than nine million people worldwide during the last six months [1, 2]. The resultant novel pneumonia coronavirus disease 2019 (COVID‐19) displays various symptoms amongst the patients including asymptomatic carriers, mild clinical manifestations and severe cases that require treatments in intensive care unit (ICU) or lead to deaths [3].

Many factors, environmental or endogenous, may have contributed to the severity of this disease but tobacco smoking has stood out first without direct research simply because both SARS‐CoV‐2 and smoking directly affect the lung. This common knowledge has led to a widely accepted assumption that tobacco smoking history may predispose the infected to severe pneumonia. However, currently there is no evidence for such environmental exacerbation, that is whether COVID‐19 patients with smoking history carry more damage in their lungs than those without smoking history.

Whether smoking history affects the prognosis for COVID‐19 remains controversial in the first place. Some studies have reported a positive association between smoking and severe COVID‐19 by analysing literature information on clinical characteristics of the disease but others have denied such an association, with odds ratios (ORs) of 0.27–12.2 [4, 5, 6, 7, 8]. As a likely reason, none of these reported studies were designed to investigate the association. It therefore remains elusive whether tobacco smoking history affects the disease progression so that a careful study is warranted.

**Fig. 1:**
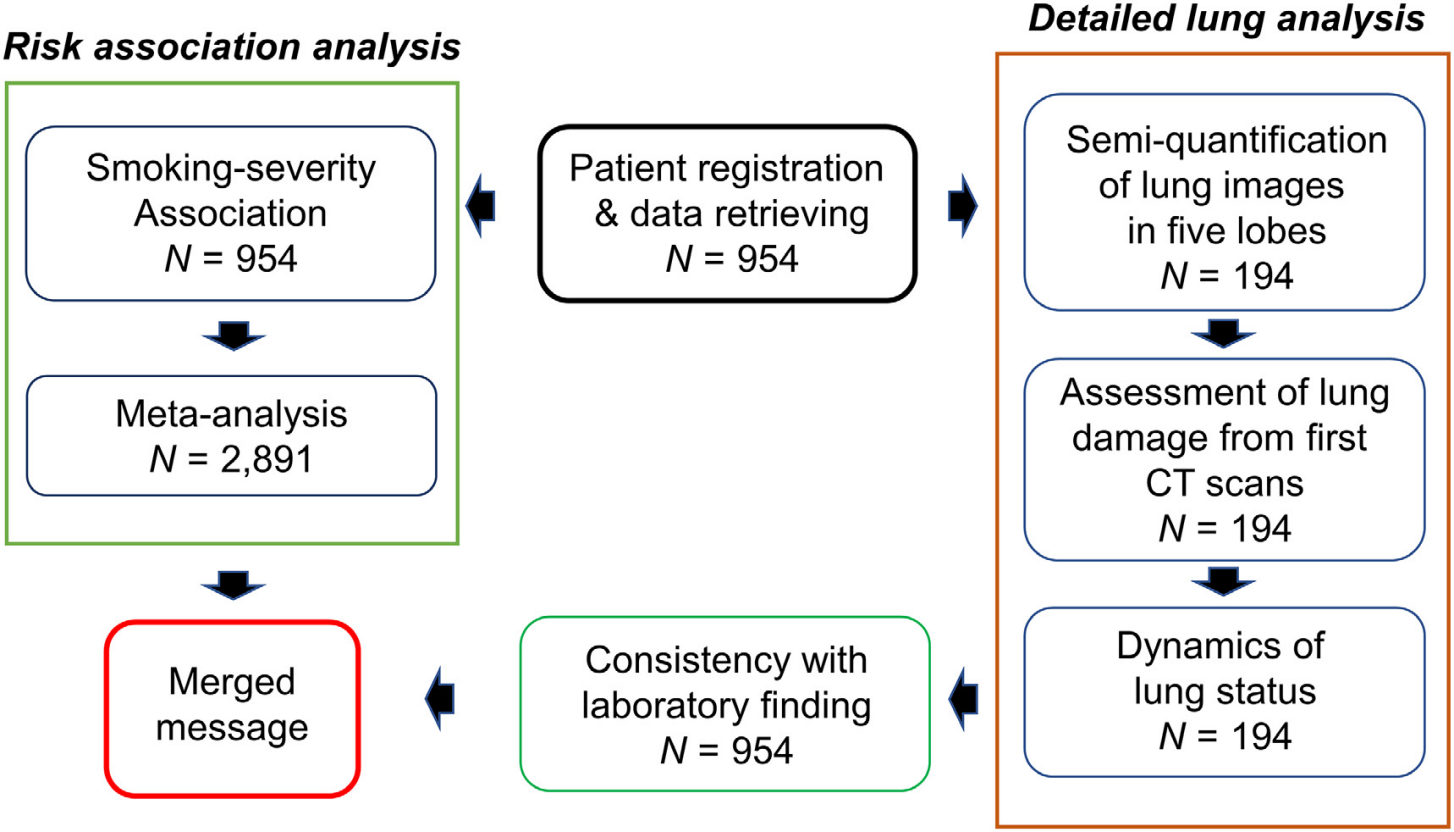
Study design.

In order to clarify the possible association and further find lung‐based supporting evidence for the association, we have carried out the first retrospective case series study focused on effects of tobacco smoking on lungs with COVID‐19, as outlined in Fig. 1, by semi‐quantitative analysis of CT chest images with lung damage score, at the initial encounter, and longitudinal changes in the score at different time‐points thereafter.

## Materials and methods

### Study design and patients

All patients were admitted and diagnosed with COVID‐19 at Wuhan Red Cross Hospital (WRCH) of China. Confirmation with reverse transcriptase–polymerase chain reaction (RT‐PCR) positive for SARS‐CoV‐2 was required for inclusion in this study. Lengths of illness and hospital stay with discharge or before death were all recorded completely. Informed consent was granted by the WRCH Committee and written consent was obtained from each patient for collection of biological samples after review and approval of the study protocol by WRCH institutional review committee (IRB) on 04/02/2020.

### Quantification of smoking history

At admission, each patient was asked about tobacco smoking history in details, including current and past, average number of cigarettes per day and years of smoking. Since background smoking, accumulated smoking and smoking year all matter [9, 10], we defined a smoker as one who had consistently smoked tobacco for at least five years and used both pack‐year and smoking year to estimate smoking history.

### Meta‐analysis

As described before [11], literature search, in both English and Chinese, went through the PubMed and the Chinese Medical Journal Network (medjournals.cn) from December 2019 to March 2020 by using the keywords ‘COVID‐19’, ‘2019‐nCov’, ‘coronavirus’, combined with ‘clinical characteristics’. The disease was defined as by the guidance issued by the Chinese National Health Committee. This period of time was chosen to match when the patients in our cohort were admitted to WRCH. If needed, authors were contacted for completeness of information. Criteria for inclusion was study on patients with both current and past tobacco smoking status, peer‐reviewed, original data, independence, severe (ICU including death cases) vs. nonsevere (non‐ICU) status. Bias risk was assessed and studies with current smoking only were excluded, resulting nine studies to be included here [3, 12, 13, 14, 15, 16, 17, 18, 19]. For the Chen N et al study, only patients with certain status/hospitalization outcomes by the time of their report were used. Meta‐analysis of data extracted from these nine studies, along with our own data, was carried out via RevMan 5.3 from Cochrane Collaboration's program [20] for various parameters including odds ratio (OR) and its 95% confidence interval (CI), heterogeneity and publication bias.

### Computed tomography (CT) chest scan

Chest was scanned with 1‐mm slice thickness CT on a Siemens SOMATOM go.Top 64 scanner (Siemens Healthineers, Suzhou, China), by using a field of view (FOV) 413 × 413 mm, tube voltage 130 kV, tube current 138 mA, pitch 0.6, reconstruction layer thickness 1.5 mm. Lung image reconstruction relied on a high‐resolution algorithm. All images were reviewed and consensus reached by two radiologists with approximately five years of experience each, followed by reporting and recording.

### CT image analysis

Each CT chest image was examined for presence of these features: (1) ground‐glass opacities, (2) consolidation, (3) laterality of ground‐glass opacities and consolidation, (4) number of lobes affected where either ground‐glass or consolidative opacities were present, (5) degree of involvement of each lung lobe in addition to overall extent of lung involvement measured by means of a ‘total severity score’ as detailed below, (6) nodules, (7) a pleural effusion, (8) thoracic lymphadenopathy (defined as lymph node size of ≥ 10 mm in short‐axis dimension), (9) airways abnormalities (including airway wall thickening, bronchiectasis and endoluminal secretions), (10) axial distribution of disease (categorized as no axial distribution of disease, central ‘peribronchovascular’ predominant disease, or peripheral predominant disease) and (11) related lung disease including emphysema and fibrosis. Other abnormalities, such as linear opacities, opacities with a rounded morphology, opacities with a ‘reverse halo’ sign, opacities with a ‘crazy‐paving’ pattern, and opacities with intralesional cavitation, were noted too. Ground‐glass opacification was defined as hazy increased lung attenuation with preservation of bronchial and vascular margins; consolidation, opacification with obscuration of margins of vessels and airway walls [21, 22]. A total of twenty features were examined and scored.

### Lung damage scoring

The right lung is slightly bigger than the left and common practice is to examine three lobes on the right and two on the left. Per cent bin‐based ‘blindly’ scoring was used to semi‐quantify lung damage. Each of the five lung lobes (left up, left low, right up, right middle and right low) was assessed for degree of involvement and classified as none (0%), minimal (1–25%), mild (26–50%), moderate (51–75%) or severe (76–100%), as reported [23]. No involvement corresponded to a lobe score of 0, minimal to a lobe score of 1, mild to a lobe score of 2, moderate to a lobe score of 3, severe to a lobe score of 4 and extensive to a lobe score of 5. An overall lung damage was estimated by combining all five lobe scores (range of possible scores, 0–20). In additional, we marked ‘0’ for absence and ‘1’ for the presence of the features mentioned above. People who gave the scores were unaware of this study. The amount of time between the initial appearance of patient symptoms (onset) and the date of admission, the dates of CT examinations and the date of discharge or death were all recorded for each patient, to calculate illness days (iDays, from onset to discharge), hospital days and rates of change in lung damage scores.

### Quantification of change in lung damage

Rate of change in damage score (*R*_s_) was estimated as

$$R_{s}=\frac{Sn‐Sm}{i\mathrm{Day}n‐iDaym}$$

where *S* was for score, *n* and *m* for iDays *n* and *m*; *i*Day was counted by setting onset as *i*Day = 0, and so on, so that *m* could be ‘0’, the onset day. There was no additional CT scan between *m* and *n*.

### Related clinical care

As part of standard care, laboratory tests of blood cell count, kidney and liver functions, and C‐reactive protein and lactate dehydrogenase levels were performed for all COVID‐19 patients at WRCH.

### Data retrieving

All information used in this study was retrieved between 04/03/2020 and 06/20/2020 from electronic health records in a standardized data collection form, which were made using data mainly at admissions, or during the hospital stays, from medical records, physicians responsible for the treatment of patients or their families to ascertain epidemiological or symptom data, all at WRCH. Information retrieving was performed and cross‐checked for accuracy by two trained physicians.

### Statistical analysis

Categorical variables were described as frequency rates and/or percentages. Bar graphs showed data in mean ± standard error of the mean (SEM). Fisher’s exact tests were used for smoking risk assessment of *P*‐values and odds ratio (OR). Algorithms implemented in Prism GraphPad (v5) were used for data analyses, including *Student*’s one‐ or two‐tailed t‐tests of means, *F*‐tests of variances, modelling of linear correlations, point‐to‐point change in individual’s score, and estimation of average Pearson correlation coefficient (*r*). *P‐*value (*P*_T_ from *t*‐test and *P*_F_ from *F*‐test) of < 0.05 was considered as statistically significant, with Bonferroni for multiple testing.

## Results

**Fig. 2:**
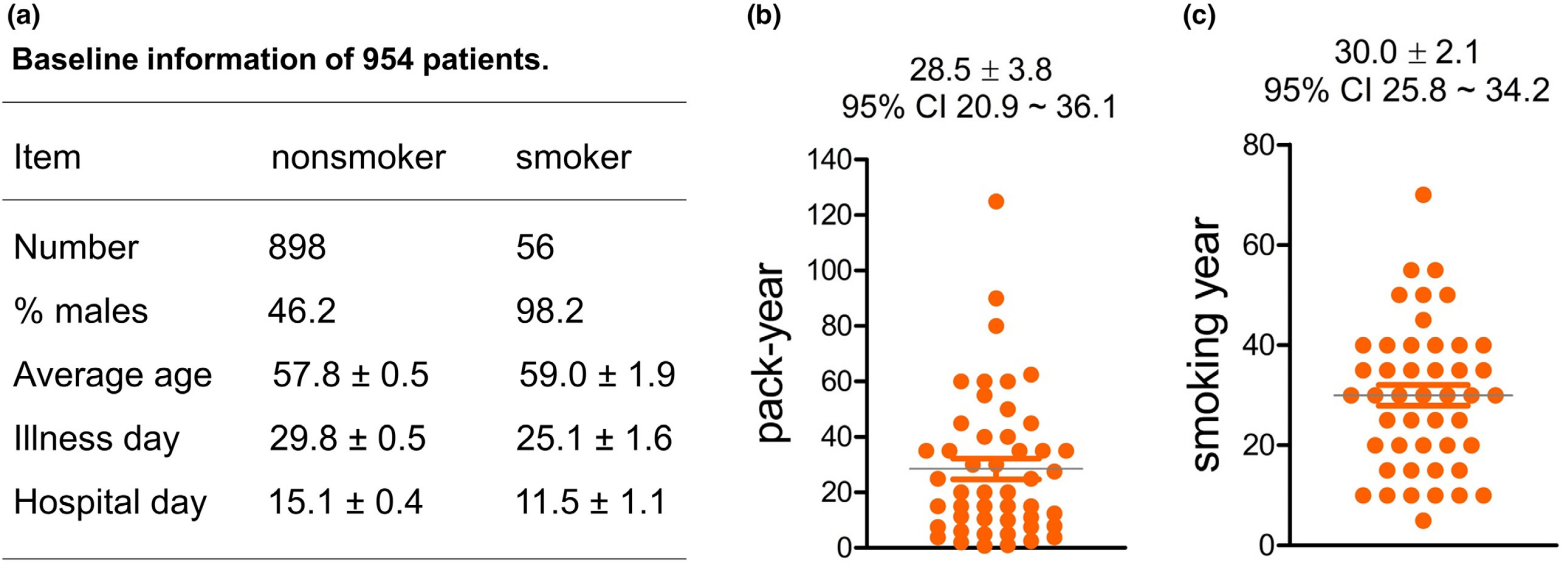
Demographic information of the study cohort. (a) Baseline information where the smokers had more % males and shorter illness or hospital days than the nonsmokers. (b) Smoking history by pack‐year for 48 patients. Eight other patients with smoking history had no specific information so were not displayed here but all were ‘current smokers’; 40 (71.4%) out of the 56 patients with smoking history were ‘current smokers’. (c) Smoking history by year of the 48 patients with smoking history. Averages are indicated on top.

This study registered 954 patients with COVID‐19 including 898 nonsmokers and 56 age‐matched smokers who were admitted to Wuhan Red Cross Hospital of China. The smoking group had bit shorter duration of illness or hospitalization than the nonsmoking group (Fig. 2a), and an average of 28.5 pack‐years or 30 years of smoking history (Fig. 2b,c).

**Fig. 3:**
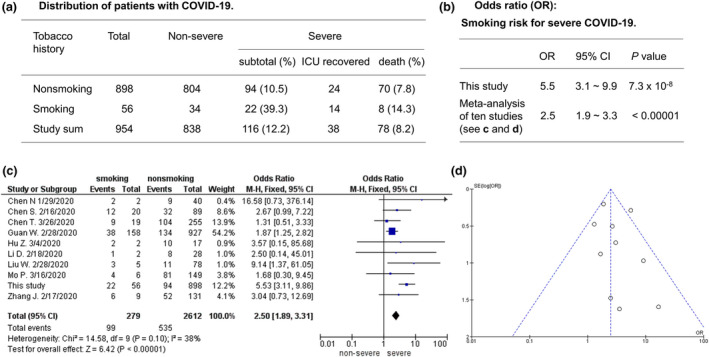
Association of tobacco smoking history with severe COVID‐19. (a) Distribution of nonsmokers and smokers in nonsevere, ICU recovery and death. (b) Statistical significance of smoking risk where two‐tailed Fisher’s exact tests were used for this study. (c) Forest plot showing 2.5‐fold risk by fixed effect model (random effect model: OR = 2.81, 95% CI 1.78–4.45, Z = 4.41, P < 0.0001). (d) Funnel plot showing no publication bias.

In this smoking cohort, smoking history increased the risk significantly for severe forms (ICU recovery and death) of this disease. We included required treatment in ICU and death as the severe forms of this disease. Epidemiologically, 39.3% of the patients with smoking history showed severe disease, comparing to 10.5% of those without smoking history (Fig. 3a), which equalled to an odds ratio (OR) of 5.5 and a significant *P*‐value of 7.3 × 10^−8^ (Fig. 3b). This finding suggested that tobacco smoking history conferred a significant risk for getting severe COVID‐19, once infected by the underlying coronavirus SARS‐CoV‐2. To validate this finding, we conducted a meta‐analysis by combining this cohort with nine published cohorts those contained patients with smoking history. The result of this meta‐analysis was an OR of 2.5 and a *P*‐value of < 0.00001 (Fig. 3c,d), supporting the association finding from this focused study. In our cohort, the association with mortality gained an OR of 1.97 with a one‐tailed *P*‐value of 0.078 by Fisher Exact tests of this cohort, which was statistically insignificant.

**Fig. 4:**
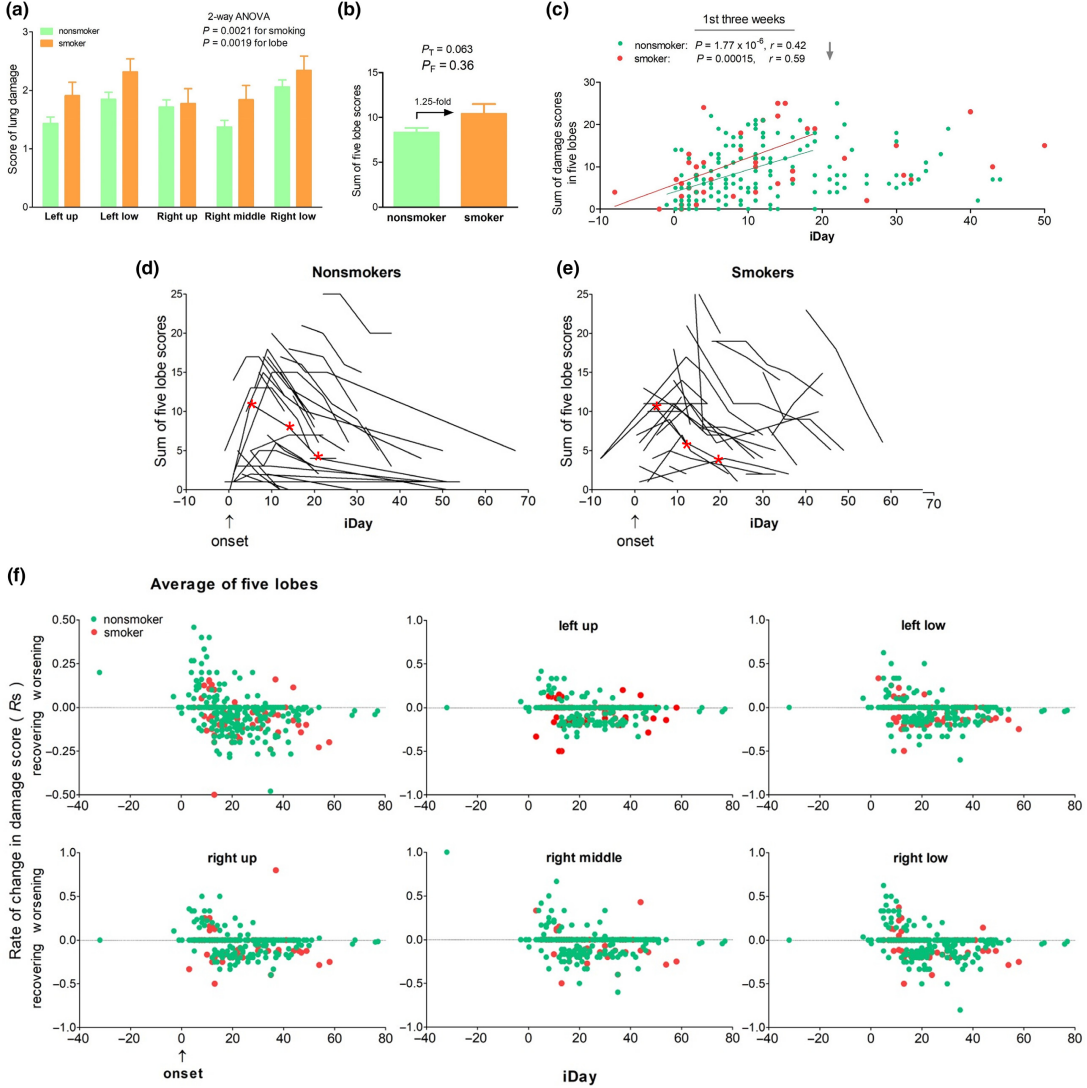
No effect of smoking history on lung damage. (a) Average scores for each of five lobes. There was neither interaction between smoking and lobe nor significant difference between nonsmoker and smoker for any of the lobe based on Bonferroni post‐tests. (b) Average of five lobes. (c) Illness day (iDay) versus total score of five lobes. Each dot represents a patient. Linear correlation statistics was for the first three weeks (grey arrow) after onset (iDay = 0, blue arrow). (d) and (e) Time course of lung (five lobes together) damage score in 95 nonsmokers and 40 smokers. Each curve or line is for one patient; iDay, illness day (0 for onset day), patients with single CT scans or outside the iDay range were not shown. Red asterisks, representative CT images from a nonsmoker and a smoker are shown in Figure S1. (f) Rate of change in lung damage score on average (upper left panel) and in each of five lobes. iDay = 0 as onset; positive score, disease progression; negative score, lung recovering; each dot represents a rate: green, nonsmoker; red, smoker.

Next, we set to search for evidence that patients with smoking history had greater lung damage than those without smoking history but disappointingly, we failed at the end. In this cohort, 194 patients each had 1–7 computerized tomography (CT) chest scans, including 150 nonsmokers and 44 smokers, collecting a total of 394 images (384 for nonsmokers and 110 for smokers). To quantitatively analyse the lung damage, we gave a score (0–5, 0 for no damage and 5 for> 75% damage) for each of the five lobes, plus scoring for 15 additional features (see Methods). A total of 10,574 scores were ‘blindly’ collected for the 194 patients. We first looked at the mean score for each of the five lobes based on each patient’s first CT scan because these represented the overall lung health around initial significant clinical manifestations. As a result, the bilateral low lobes had higher scores than other lobes, and by group comparison, the smokers had consistent higher scores than the nonsmokers based on two‐way ANOVA statistics, but not by t‐tests for any of the lobes or for the summed score of five lobes (Fig. 4a,b). The negative results remained when males only were considered (data not shown). At a detailed level, the first CT scans did reveal a significant difference amongst 20 single features: air bronchogram score was higher on average in the smoking group than in the nonsmoking group (0.43 vs 0.19: *P*_T_ = 0.0012).

To consider disease stage of the CT scans, summed scores were displayed based on illness days (iDays). This display showed that within three weeks of onset, the scores increased, as the infection progressed, and the slope was similar between the two groups (Fig. 4c). Since the first cans occurred at different stages of the disease, we then used within subject‐control by investigating score dynamics at different time‐points for each patient. As Fig. 4d,e shows, within 12 days of onset, most scores increased for disease progression and then, the scores dropped for lung recovering, with no difference between the two groups (see CT images in Figure S1 in Supplementary Content for same recovery rate between a representative nonsmoker and a representative smoker). The lack of difference in lung health between nonsmokers and smokers was verified in detail by similar rates of change in damage score (*R*s) for each of the lobes in individual patients (Fig. 4f). These data suggested continuously that the smoking history had little effect on the COVID‐19 lungs, which had been supported by insignificant F‐test results on average lung score from the first CT scans (Fig. 4b).

**Fig. 5:**
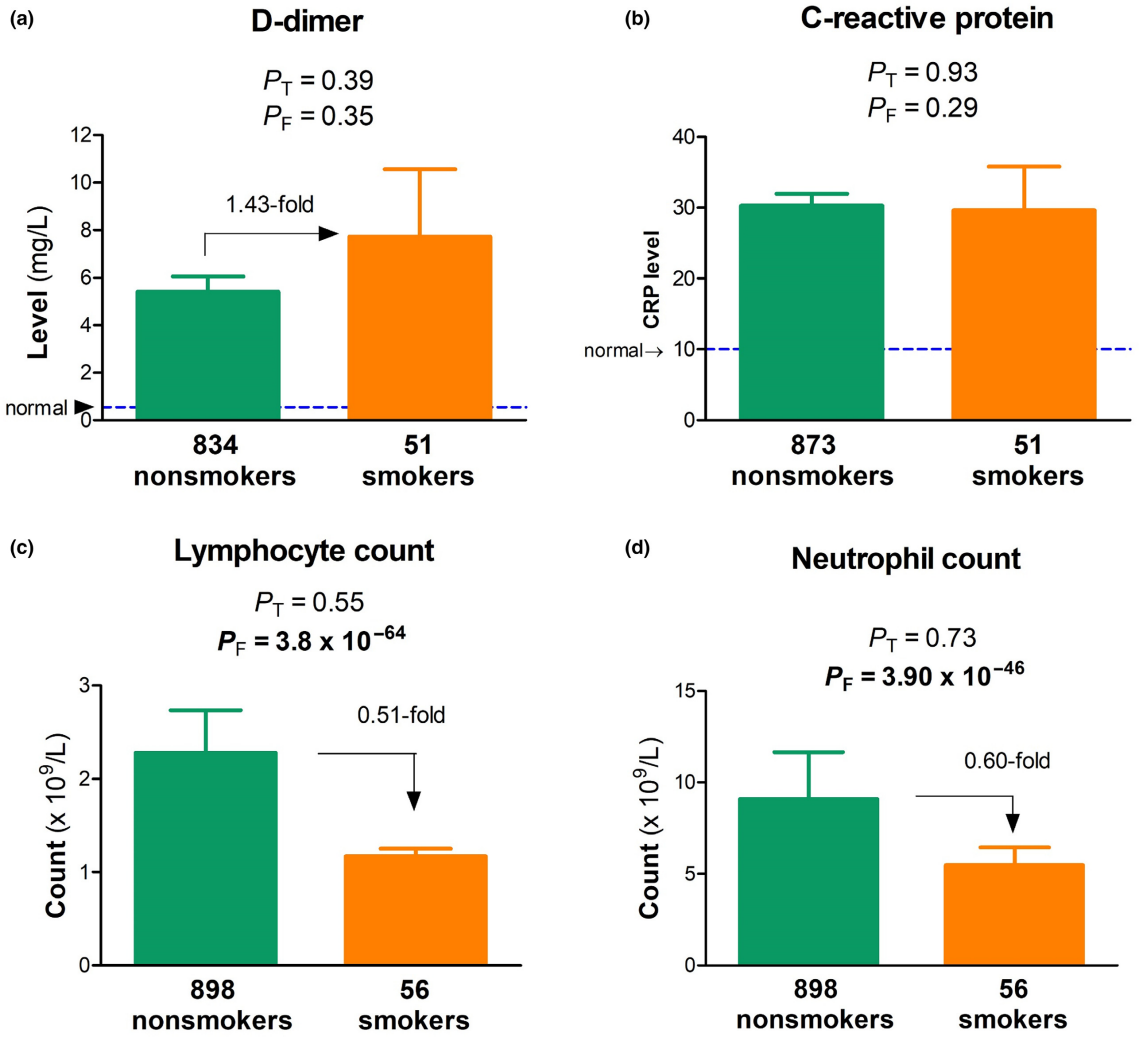
Indirect effect of tobacco smoking history on laboratory findings.(a) D‐dimer, (b) CRP, (c) lymphocyte count and (d) neutrophil count in nonsmokers (green) versus smokers (brown), with numbers of patients indicated; *P*_T_ and *P*_F_, *P*‐values from t‐tests and F‐tests; bold, statistically significant.

In order to have a comprehensive understanding of any smoking effects, we then compared more than 20 laboratory measures between the two groups but found no significant differences either outside normal ranges. High D‐dimer and C‐reactive protein (CRP) levels are typical of this disease but neither t‐tests nor F‐tests identified a difference (Fig. 5a,b). Interestingly, results from F‐tests pointed to a significant difference in covariance for immune cell density (lymphocyte and neutrophil counts; Fig. 5c,d).

## Discussion

For the first time, this study carried out a thorough analysis of smoking epidemiology, helping clarify that tobacco smoking confers a significant risk for COVID‐19 to progress to severe stages. An advantage of this single‐site clinical investigation was the same unbiased and defined measures applied to all confirmed patients, which is critical for a reliable result [24]. However, despite same standards used, extensive and intensive imaging analyses failed to find significant differences in lung health between the smoking and nonsmoking groups. This negative finding was surprising, suggesting that SARS‐CoV‐2 infection ‘overwrite’ any effect of smoking on the lungs probably because the lungs bear the brunt of the infection.

Our smoking‐focused study finds a large and positive effect size of smoking history for disease severity, by utilizing standardized questionnaire and clear criteria for smoking history and disease stages. With different results, other epidemiology reports on smoking effects might have study design issues of inconsistency, including focus on ‘current’ smokers only, patients without hospitalization outcomes yet, lack of a criterium for smoking history or ‘survivors’, as have been recognized [24]. Few meta‐analyses with those reports have been reported but the results could be spurious if the individual studies used didn’t have a clean, standardized design. These inconsistencies in study design may explain why results from the reported meta‐analyses are heterogenous: smaller ORs, lack of association or even recognizing smoking as a protective factor [5, 8]. For the same reason, our meta‐analysis didn’t represent the most reliable or comprehensive either although we carefully selected the nine studies based on their clinical descriptions. In addition, our results, like others [8], didn’t find a statistically significant association with mortality, suggesting that smoking itself be not a critical determinant of mortality in this disease but this suggestive finding will need more cases to evaluate.

Smoking history has little effect on coronaviral infection of the lungs. We chose to include cases with both current and past smoking history because the effects are postulated to be accumulative [9, 10]. Despite the accumulation, little effects were found on the lungs based on 20 different damage measures. Previous studies have shown that emphysema is the key result of the smoking lung [25, 26]. Emphysema can also be a result of coronaviral infection of the lungs [27, 28, 29]. Many COVID‐19 patients are often infected with germs causing pneumonia – and that this in turn can lead to lung damage of the emphysema type. These findings suggest that the later result ‘overwrite’ the former result, which may explain the lack of difference in lung damage between the two groups. Besides, air bronchogram can also be a result of the infection [23, 30] and our finding suggests that smoking history may facilitate the formation of air bronchogram in lungs with COVID‐19.

Where did the smoking risk come from? Our study failed to find a solid answer with the negative results in lung damage. One explanation is that COVID‐19 severity is not determined by the lung damage alone, given the fact that COVID‐19 is a multiorgan damage disease, possibly due to systemic immune overreaction [31, 32]. Our laboratory results on density of immune cells uncover for the first time significant difference in covariance between the two groups and this finding may indicate an effect of smoking history on the immune system instead. This rationale is consistent with the view that smoking history suppresses the immune system to confer the risk [33]. This view also explains the smoking‐associated systemic and slight increase in the damage scores from the first scans (Fig. 4a) but not necessarily in the right lung although COVID‐19 affects the right lung preferentially [34].

Prevalence of smoking is low amongst patients with COVID‐19. More than 25% of the Chinese population are exposed to tobacco smoking [35, 36]. In this cohort, the prevalence is only less than 6% in the patients, consistent with what has been reported for 5960 different patients [37], suggesting that our cohort was representative of COVID‐19 patients. One explanation is that smokers usually keep social distancing, which is effective to minimize the smell and smoke exposure in the public [38]. This ‘natural’ social habit may have helped and reduced the coronavirus transmission to the smokers, resulting in the low prevalence. This low prevalence, on the other hand, has made it more challenging to study smoking effects on COVID‐19 in an unbiased manner and large cohorts.

Limitations of this retrospective study included limited sample size, especially for the smoking group, limited ethnicity and incomplete collection of CT images. This was a clinical association analysis, not a clinical trial, so that it didn’t reveal causality. Gender was not controlled in all analyses since most of smokers were males. A control for receptor expression levels was not considered for disease severity. Nicotine‐related vascular dynamics was not followed up yet. The large OR obtained in this cohort thus needs replication by additional well‐designed studies in larger cohort and different ethnicities.

## Conclusion

Tobacco smoking confers a significant risk for severe COVID‐19 which leads to ICU necessity or death. However, it is unlikely the lung that mediates the risk. Rather, the great severity may more likely be a result of immune suppression by accumulated smoking.

## Conflict of interest

None of the authors declare conflict of interest.

## Author contribution

**Zhicheng Lin** and **Nian Xiong** conceived the study for different sections (contributors): smoking vs nonsmoking grouping (**Jingwen Li, Nian Xiong**), iDay, hDay information collection, verification, smoking epidemiology and smoking amount data (**Qing Zhang, Na Li, Xi Fang**), laboratory results and other data retrieving or statistical t‐tests analysis (**Qing Zhang, Xi Fang, Jingwen Li, Nian Xiong**), CT scan: imaging collection, analysis, scoring and data sheet preparation (**Xi Long, Shaoping Hu, Nian Xiong**), CT imaging methods section drafting (**Jingwen Li, Xi Long, Shaoping Hu, Nian Xiong**), CT image selection and preparation for a smoker and a nonsmoker (**Xi Long, Shaoping Hu, Nian Xiong**), other data analyses and manuscript drafting (**Zhicheng Lin**); and manuscript finalization (**Zhicheng Lin, Nian Xiong, Jinghong Li, Barbara Fedorova**). Supervision: **Zhicheng Lin** and **Nian Xiong**. All authors declare no competing interest.

[Correction added on 20 January 2021, after first online publication: the author names have been updated in the author contribution statement.]

## Funding

This work was supported by grants 2016YFC1306600 and 2018YFC1314700 from the National Key R&D Program of China, 81873782 from the National Natural Science Foundation of China (NX), and the U.S. National Institutes of Health grant DA021409 (ZL).

## Supporting information


**Figure S1.** CT images: similar recovery ability of COVID‐19 lungs between a representative nonsmoker and a representative smoker.
