# Supplementary figures and images for: Tobacco smoking confers risk for severe COVID‐19 unexplainable by pulmonary imaging
Source: J Intern Med. 2020 Dec 3;289(4):574–83. doi: 10.1111/joim.13190 (PMC7753648; doi:10.1111/joim.13190)

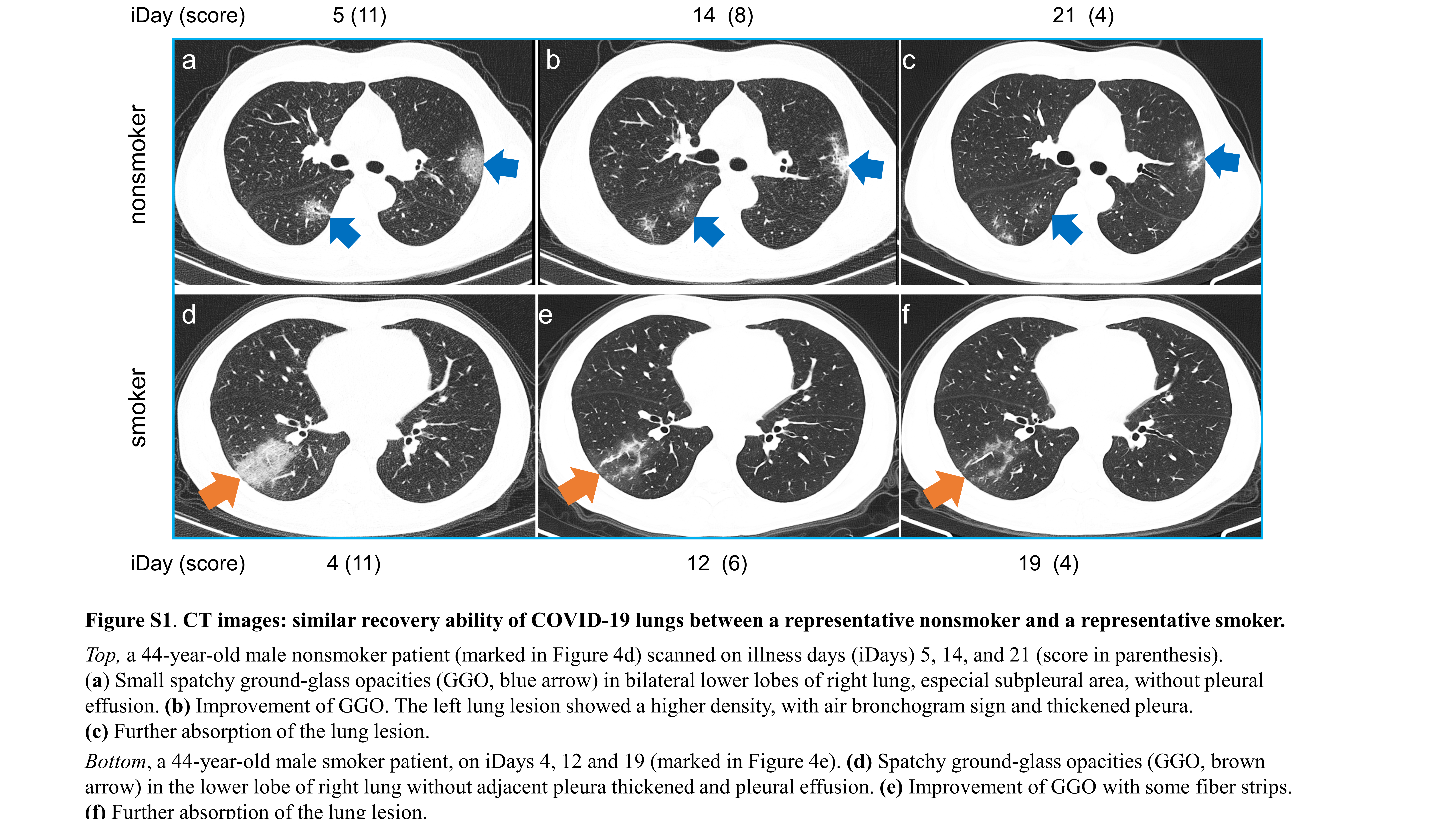

Supplement: Supplementary file 1 — Figure S1. CT images: similar recovery ability of COVID‐19 lungs between a representative nonsmoker and a representative smoker. [file JOIM-289-574-s001.tif]
